# A Novel Tree Shrew Model of Chronic Experimental Autoimmune Uveitis and Its Disruptive Application

**DOI:** 10.3389/fimmu.2022.889596

**Published:** 2022-05-27

**Authors:** Kaijiao Hu, Longbao Lv, Hui Huang, Guangnian Yin, Jie Gao, Jianping Liu, Yaying Yang, Wenxin Zeng, Yan Chen, Ni Zhang, Feiyan Zhang, Yuhua Ma, Feilan Chen

**Affiliations:** ^1^ Laboratory Animal Center, Chongqing Medical University, Chongqing, China; ^2^ Chongqing Engineering Research Center for Rodent Laboratory Animals, Chongqing, China; ^3^ Laboratory Animal Center, Kunming Institute of Zoology, Chinese Academy of Sciences, Kunming, China; ^4^ Department of Clinical Laboratory, the Second Affiliated Hospital of Army Medical University, Chongqing, China; ^5^ Department of Pathology, Chongqing Medical University, Chongqing, China; ^6^ The First Affiliated Hospital of Chongqing Medical University, Chongqing Eye Institute, Chongqing, China

**Keywords:** experimental autoimmune uveitis (EAU), chronic, tree shrew, differentially expressed gene (DEG), metabolic pathways, microglia, T-cell response, regulators of G-protein signaling 4 (RGS4)

## Abstract

**Background:**

Previous studies have established several animal models for experimental autoimmune uveitis (EAU) in rodents without the fovea centralis in the human retina. This study aimed to develop and explore the application of a novel EAU model in tree shrews with a cone-dominated retina resembling the human fovea.

**Methods:**

Tree shrews were clinically and pathologically evaluated for the development and characteristics of EAU immunized with six inter-photoreceptor retinoid-binding proteins (IRBPs). IRBP-specific T-cell proliferation and serum cytokine of tree shrews were evaluated to determine the immune responses. Differentially expressed genes (DEGs) were identified in the eyes of tree shrews with EAU by RNA-sequencing. The disruptive effects of the DEG RGS4 inhibitor CCG 203769 and dihydroartemisinin on the EAU were investigated to evaluate the potential application of tree shrew EAU.

**Results:**

IRBP_1197–1211_ and R14 successfully induced chronic EAU with subretinal deposits and retinal damage in the tree shrews. The immunological characteristics presented the predominant infiltration of microglia/macrophages, dendritic cells, and CD4-T-cells into the uvea and retina and pathogenic T helper (Th) 1 and Th17 responses. The subretinal deposits positively expressed amyloid β-protein (Aβ), CD8, and P2Y purinoceptor 12 (P2RY12). The crucial DEGs in R14-induced EAU, such as *P2RY2* and *adenylate cyclase 4 (ADCY4)*, were enriched for several pathways, including inflammatory mediator regulation of transient receptor potential (TRP) channels. The upregulated *RGS4* in IRBP-induced EAU was associated with mitogen-activated protein kinase (MAPK) activity. RGS4 inhibition and dihydroartemisinin could significantly alleviate the retinal pathological injuries of IRBP_1197-1211_-induced EAU by decreasing the expression of CD4 T-cells.

**Conclusion:**

Our study provides a novel chronic EAU in tree shrews elicited by bovine R14 and tree shrew IRBP_1197-1211_ characterized by retinal degeneration, retinal damage with subretinal Aβ deposits and microglia/macrophage infiltration, and T-cell response, probably by altering important pathways and genes related to bacterial invasion, inflammatory pain, microglial phagocytosis, and lipid and glucose metabolism. The findings advance the knowledge of the pathogenesis and therapeutics of the fovea-involved visual disturbance in human uveitis.

## Introduction

Worldwide, uveitis is a group of common blinding intraocular inflammatory disorders, some of which arise with an unknown infectious trigger and are frequently associated with immune reactions to unique retinal proteins (1, 2). Retinal folds, photoreceptor cell damage, and inflammatory infiltration into the eyeball are primary features of autoimmune uveitis and lead to vision loss in humans. Although rods outnumber cones, human vision mostly relies on cone photoreceptors since cones play an important role in sharp chromatic vision and spatial acuity (3). In particular, the human fovea, a specialized region of the central retina, shows the highest density of cones and is essential for human vision (4). Despite numerous research efforts, the etiology of uveitis remains unclear, and effective therapies need to be further identified. Experimental autoimmune uveitis (EAU) in nocturnal rodents with sparse cone is utilized in research to investigate human diseases, but such animal models cannot reflect the effect of fovea on vision loss in human disorders, so it is necessary to develop a variety of animal models to represent the distinct aspects and diverse findings of uveitis in humans.Tree shrews are a diurnal species with cone-dominated retina and immune systems similar to those of both primates and humans (5, 6). They have been widely used as a model animal for studying glaucoma, myopia, refractive development, and central visual processing and are becoming a crucial model animal in vision research (7–9).

Here, we developed a novel tree shrew model of uveitis elicited by bovine interphotoreceptor retinoid-binding protein (IRBP) R14 (BR14, aa 1169–1191) and tree shrew IRBP_1197–1211_ characterized by retinal folding and damage with subretinal amyloid β-protein (Aβ) deposits and microglia/macrophage infiltration as well as T-cell response. Meanwhile, the differentially expressed gene (DEG) characteristics in IRBP-induced and R14-induced EAU are involved in the breakdown of the blood-ocular barrier, inflammatory pain, and microglia phagocytosis. The inhibitor of DEG regulators of G-protein signaling 4 (RGS4) and the Chinese herb derivative dihydroartemisinin (DHA) had a protective effect against IRBP-induced EAU by reducing the expression of CD4 cells in the eyes after treatment.

## Materials and Methods

### Animals and Paraffin-Embedded Human Eye Tissue

Chinese tree shrews (*Tupaia belangeri chinensis*) were purchased from the Kunming Institute of Zoology (KIZ), Chinese Academy of Sciences (CAS), and the Institute of Medical Biology Chinese Academy of Medical Sciences and housed in laboratory animal facilities at Chongqing Medical University. They were freely given access to chow and water. All of the procedures were carried out in compliance with the guidelines of the National Institutes of Health and the ARVO Statement and the ARRIVE for the Use of Animals in Ophthalmic and Vision Research regarding the animals’ care and use (10). The protocol for the study was approved by the Ethics Committee of Chongqing Medical University (permit no. 2021031). Paraffin-embedded human eye tissues diagnosed as uveitis by a pathologist were obtained from the Department of Pathology, Chongqing Medical University.

### Immunization and Treatment of EAU in Tree Shrews

For induction of EAU, tree shrews 4–8 months of age were subcutaneously immunized at the base of the tail and both thighs with different immunized conditions (**Table 1**) according to previous studies (11). Briefly, peptides from 50 μg to 800 μg of four tree shrew R14 (aa 1169–1191) and R16 (aa 1177–1191), IRBP_1197–1211_, IRBP_1041–1071_, and two bovine R14 and R16 (China Peptides Co., Ltd., Shanghai, China) were emulsified in a 200-μL emulsion in complete Freund’s adjuvant (CFA, St Louis, MO,USA) containing or without *mycobacterium tuberculosis* (H37RA, ATCC 25177; American Type Culture Collection, Manassas, VA, USA). Simultaneously, pertussis toxin (PTX, Sigma-Aldrich, St. Louis, MO, USA) in 200 μL was intraperitoneally injected into some animals. Four additional groups of male and female tree shrews aged 7–8 months were immunized with 800 μg of tree shrew IRBP in a 200-μL emulsion in CFA containing 500 μg of *mycobacterium tuberculosis (TB)* and an additional 1000 ng of PTX to investigate the protective effect of RGS4 inhibition and dihydroartemisinin on the pathological lesions of EAU in tree shrews. Six tree shrews were treated with 16 mg/kg of dihydroartemisinin in 100-µL or with the same volume of corn oil intraperitoneally every other day from day 0 to day 25/45 day postimmunization. Another six tree shrews were treated with 0.137mg/kg of CCG 203769 in 100-µL corn oil intraperitoneally every day from day 0 to day 2 postimmunization.

**Table 1:** Immunization conditions of different retinal antigens with various dosages.

Type of retinal antigen	Sequence	Dosage ofAg (µg)	TBX(mg/ml)	PTX(ng)
Tree shrew R14	PTARSVGAADGTSWEGVGVVPHV	100	1	0
		800	5	1000
Tree shrew IRBP_1197-1211_	GAADGTSWEGVGVVP	50	5	1000
		300	3.5–5	500
		400	5	1000
		800	5	1000
Tree shrew R16	ADGTSWEGVGVVPHV	50	5	0
		500	3.5	2000
Tree shrew IRBP_1041–1071_	GYLRFDMFGDCELLTQVSELLVEHIWKKIVH	300	1	500
Bovine R14	PTARSVGAADGSSWEGVGVVPDV	50	5	1000
		300	3.5–5	1000
		400	5	1000
		800	5	1000
Bovine R16	ADGSSWEGVGVVPDV	50	5	0
		100	1	0

### Clinical and Histological Evaluations

The immunized tree shrews were checked two or three times per week to observe the clinical signs from day 5 postimmunization using a slit-light scope and a funduscope. Then, immunized animals were euthanized on days 25–27, 40–45, or a humane endpoint postimmunization. Eyes of the immunized tree shrews were harvested and fixed in 4% glutaraldehyde for one hour, then immediately passed to 10% buffered formaldehyde for histopathology of the EAU. Paraffin-embedded eye tissues were sliced into 4-μm sections for standard hematoxylin and eosin (H&E) staining. Clinical and histopathological grading in the animals was performed in a masked manner on a scale from 0 to 4 points following the previously reported criteria (12, 13).

### T Cell Proliferation

Spleens from the EAU tree shrews in the experimental and control groups were collected on day 25. Although it has been reported that nylon wool enrichment of T cells can change their activity after cross-linking the T cell receptor (14), it is reliable for antigen-primed T cell proliferation assays according to various tumor and immune studies (15, 16).Therefore, enrichment of antigen-presenting cells and T-cells in the spleens and lymph nodes was performed by passage through a nylon wool column, following the methods described in a previous study (17). Next, 1×10^5^ APC cells and 4×10^5^ T-cells were co-cultured in a 96-well plate with final concentrations of 0, 10, and 20 μg/mL of IRBP-specified antigens at 37°C in 5% CO_2_ for 72 hours. The tetrazolium salt MTT was added, and the response was detected using an automatic microplate reader (Thermo Fisher Scientific Inc., Waltham, Massachusetts, USA) at a wavelength of 490 nm. The proliferation reaction was expressed as the mean optical density (OD) ± standard error of the mean (SEM) of the triplicate assays.

### Cytokine Analysis by Flow Cytometry

Peripheral venous blood was drawn into a heparinized tube from tree shrews immunized with IRBP_1197–1211_ or BR14 with or without CCG 203769 and DHA on day 25 and day 40 postimmunization in control tree shrews. Serum was collected from tree shrews by centrifugation for cytokine detection. Serum was utilized for flow cytometric analysis. Flow cytometry was performed with a FACS Canto II (BD Biosciences, San Jose, CA, USA). Data were analyzed with the FlowJo software (TreeStar, Ashland, OR, USA).

### RNA Extraction

A total of nine eyes from IRBP-induced EAU, BR14-induced EAU and healthy age-matched control tree shrews were collected to analyze the DEG profiles on day 26 postimmunization. The total RNA was extracted from the eyes using TRIzol^@^ reagent (Invitrogen, Carlsbad, CA, USA).

### Gene-Expression Profile Analysis

RNA-sequencing (RNA-seq) was carried out by Origingene Biomedical Technology Co., Ltd. (Shanghai, China). The mRNA was enriched from the total RNA and reverse-transcribed into complementary DNA, which was then sequenced on an Illumina Hiseq X-Ten (Illumina, Inc., San Diego, CA, USA). Raw data in the FASTQ format were analyzed with a quality evaluation using FastQC version 0.11.4 (Illumina, Inc., San Diego, CA, USA) and were filtered to obtain clean reads. The reads were mapped to the *Tupaia chinensis* genome (https://www.ncbi.nlm.nih.gov/genome/?term=txid246437[orgn], TupChi_1.0) using HISAT2 to obtain the total mapped reads or fragments (18). Fragments per kilobase million (FPKM)-mapped read values were utilized to calculate the expression of the genes in each specimen. Genes with |log2 (fold change) | > 1 and a false-discovery rate (FDR) < 0.05 were chosen as DEGs using the edger package for R v 3.24 (R Foundation for Statistical Computing, Vienna, Austria) (18, 19). New genes or new transcripts were identified from assembled reads with stringTie. Functional information of genes or transcripts was annotated according to the databases in NR, Swiss-Prot, Pfam, STRING, GO, and KEGG. The enrichment analysis of the GO terms and pathways was performed by traditional singular enrichment analysis. Fisher’s exact test was performed for the enrichment *P* value calculation.

### Quantitative Polymerase Chain Reaction

To validate the DEG library, the expressions of four DEGs of eight eyes and the endogenous control gene of actin were elucidated by quantitative polymerase chain reaction (qPCR) in triplicate on a CFX 96 real-time PCR detection system (Bio-Rad Laboratories, Hercules, CA, USA). The primers are listed in **Table S1**. The 2^−ΔΔCt^ method was utilized to determine the relative gene expressions.

### Statistical Analysis

The experiments were performed two or three times to validate the experimental data. Statistical analysis was conducted using SPSS version 17.0 for Windows (IBM Corporation, Armonk, NY, USA). Clinical and histopathological scores were compared between two groups using the Mann–Whitney test or among three groups using the Kruskal–Wallis test. An unpaired Student’s *t*-test for two groups or one-way analysis of variance for three or more means was used for analysis. The level of statistical significance was set at P < 0.05.

## Results

### R14 and IRBP_1197–1211_ Induce Ocular Inflammation With Chronic Clinical Findings in Tree Shrews

**Figure 1:**
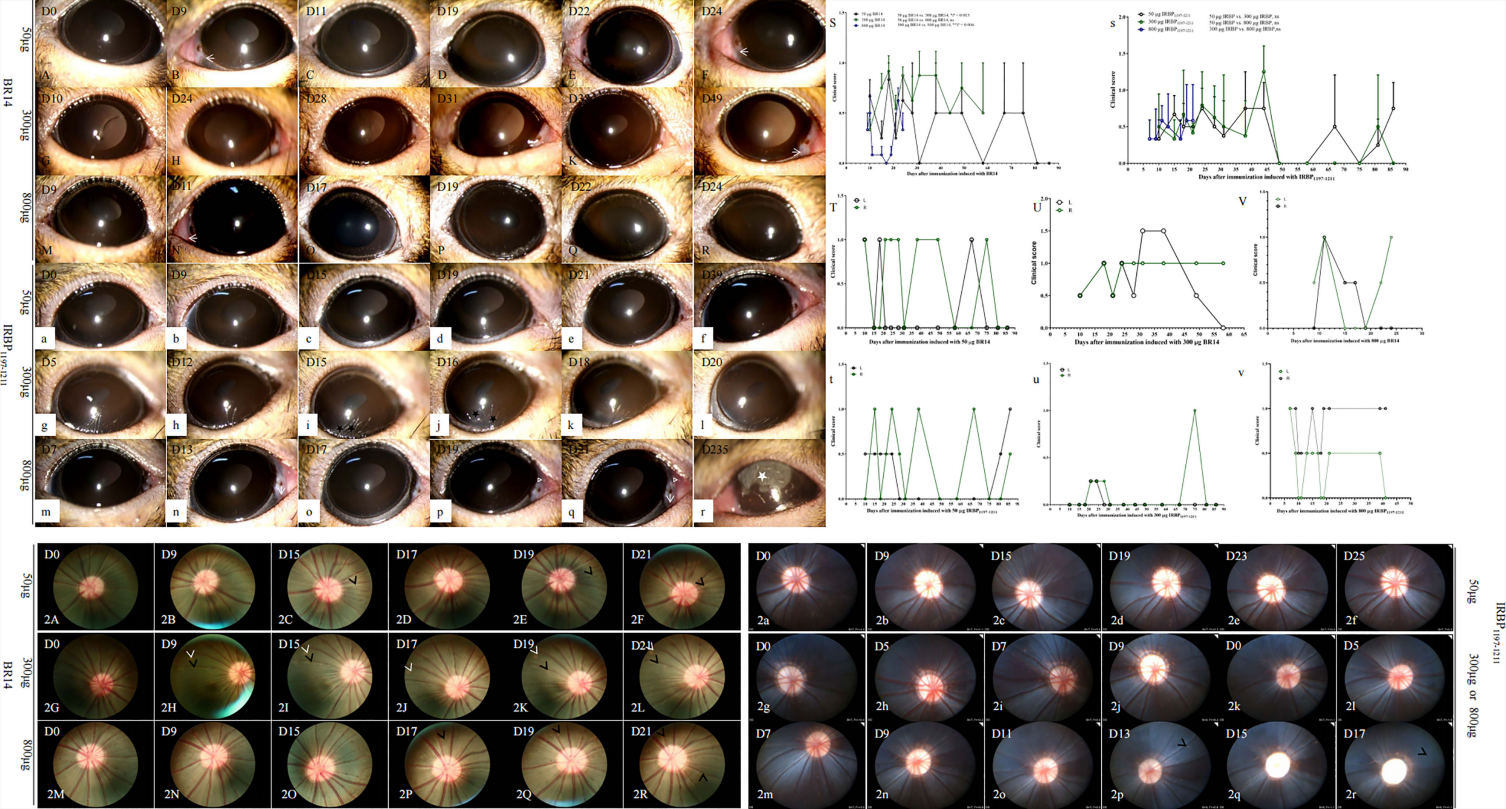
Representative clinical signs and scores of tree shrew EAU induced by R14 or IRBP_1197–1211_. Major clinical signs including conjunctival hyperemia, ciliary injections (arrow), whitish hypopyon spots (triangle), corneal ulcers (black star), and corneal edema (white star), were observed in tree shrews immunized with different dosages of BR14 **(A–R)** or IRBP_1197–1211_ **(a–r)**. Average clinical scores per eye of EAU are shown from groups immunized with either R14 **(S)** or IRBP_1197–1211_ **(s)** in CFA. A chronic and/or relapsing course of three representative animals is shown, specifically the time courses of clinical disease from tree shrews immunized with 50 µg of BR14 **(T)**, 300 µg of BR14 **(U)**, 800 µg of BR14 **(V)**, 50 µg of IRBP_1197–1211_ **(t)**, 300 µg of IRBP_1197–1211_ **(u)**, and 800 µg of IRBP_1197–1211_ **(v)**. The curves show the inflammation of eyes checked by slit-lamp biomicroscopy according to previously described criteria. Green circle, right eye; black circle, left eye; D, day post-immunization. **(2A–2R)** indicates fundus pictures of EAU in tree shrews immunized with BR14 or IRBP_1197–1211_. Retinal vessels with mild cuffing (black arrow), retinal degeneration (white arrow), generalized changes in reflectivity or hyper-reflectivity, pale optic nerve, opacification, and vascular attenuation were observed during fundus examination in the late stage of tree shrew EAU induced with 300 μg of IRBP_1197–1211_ **(2g–2j)** and 800 μg of IRBP1197–1211 **(2k–2r)**. D, day post immunization.

**Figure 2:**
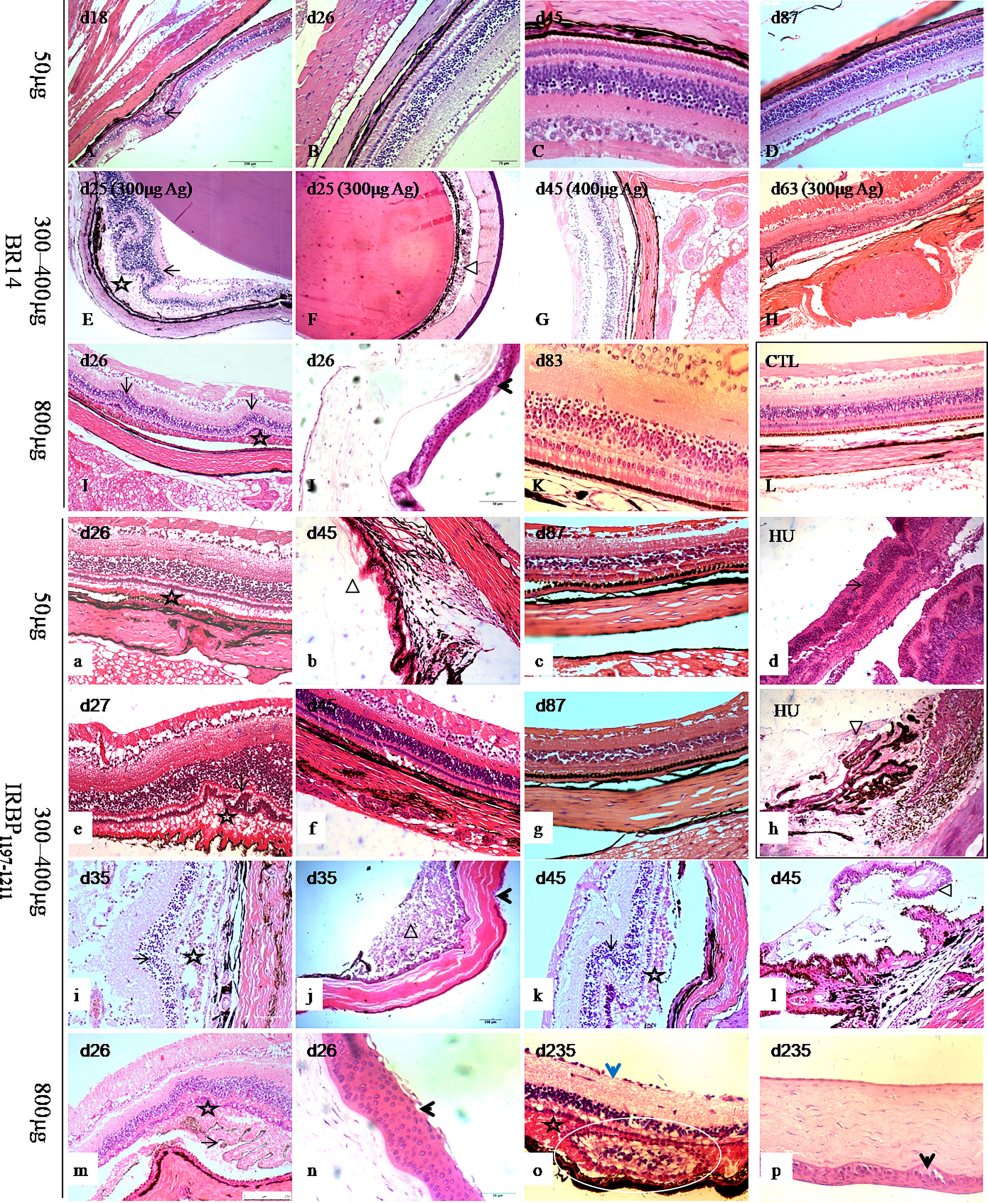
Histological pictures of human uveitis and EAU in tree shrews immunized with BR14 or IRBP_1197–1211_. The retinas of tree shrews with EAU **(A-K, a-c, e-g, i-p)** were characterized by retinal folds (long arrow) and detachments, forms of subretinal amorphous eosinophilic deposits (stars), inflammatory exudation (triangle), and corneal lesions (short arrows) in the early, peak, and late phases. Atrophy (short blue arrow) and abnormal structures (white circle) in the retinal layer with thin photoreceptor cells, bipolar cell layers, and ganglion cell layers were observed during the late stage. **(L)** shows the eye tissue of healthy control tree shrews. HU shows human uveitis in the retina and ciliary body **(d, h)**. **(b, h)** show the changes in the retina and ciliary body. D, day postimmunization.

To establish EAU in the tree shrew model, adult female and male animals were subcutaneously immunized with different dosages of R14, R16, and IRBP_1197–1211_ peptides from bovine and tree shrew sources (**Table 1**). The ocular anterior and posterior segments were observed two or three times per week at day 5 postimmunization. Tree shrews immunized with bovine R14 and IRBP_1197–1211_ from tree shrews developed chronic EAU, with onset occurring between days 5–15 and a peak happening between days 9–25, followed by varying periods of remission postimmunization (**Figure 1**). No obvious clinical findings were observed in tree shrews immunized with another retinal antigen. Conjunctival hyperemia and whitish hypopyon spots (**Figure 1**, triangle), ciliary injection (**Figure 1**, arrow), corneal edema, and corneal ulceration (**Figures 1i, j** stars) were observed by slit-lamp microscopy at days 5–15 postimmunization with 50, 300, and 800 µg of bovine R14 or IRBP_1197–1211_, lasting for a few days before disappearing, which was then followed by varying periods of remission and relapse (**Figures 1A–R, a–r**). The clinical scores of eyes in 300-µg and 800-µg BR14–induced-EAU were higher than those in 50-µg BR14–induced-EAU (**Figure 1S**). There was no significant difference between the different dosages of IRBP-induced EAU (**Figure 1s**). The course of EAU tree shrews immunized with bovine R14 or IRBP_1197–1211_ was chronic and/or relapsing (**Figures 1S–V, s-v**). The recurrences varied between individual eyes and animals, and the incidences of recurrence were 100% (6/6), 33% (2/6), 100% (4/4), 11% (1/9), 50% (4/8), and 50% (4/8), respectively, in the 50-µg-BR14–induced, 300-µg-BR14–induced, 800-µg-BR14–induced, 50-µg-IRBP_1197-1211_–induced, 300-µg-IRBP_1197-1211_–induced, and 800-µg-IRBP_1197-1211_–induced tree shrews after an extended observation period of 3–13 weeks postimmunization. The ratio of occurring death was 7 in 35 animals in BR14-induced animals and 7 in 46 in IRBP_1197-1211_–induced animals during the development of disease. Mild vascular cuffing (**Figure 1**, short white arrow), retinal degeneration (**Figure 1**, short black arrow), a generalized change in reflectivity or hyper-reflectivity, pale optic nerve, opacification, and vascular attenuation were observed during fundus examination during at late stages of tree shrew EAU (**Figures 1, 2j, 2n–r**).

### Chronic and Progressive Pathological Damages of Eyes in R14-Induced and IRBP-Induced EAU Tree Shrews

The main histopathological changes (**Figure 2A-K, a-c, e-g, i-p**) involved inflammatory infiltration (**Figure 2**, triangle) into the conjunctiva, cornea, anterior chamber, ciliary processes (**Figure 2l**, triangle), choroid, and retina (**Figures 2E, i**) as well as retina lesions (**Figure 2**, stars and arrow) in the 50-μg, 300-μg, 400-μg, and 800-μg Ag–induced tree shrews. The retinas of EAU tree shrews were characterized by retinal folds (**Figure 2**, black arrow) and detachments, retinal pigment epithelium folds, loss of the photoreceptor outer segments, formation of subretinal amorphous eosinophilic deposits (**Figure 2**, stars), and abnormally long photoreceptor cell nuclei at the early and peak phases of the disease. However, the retina was more so characterized by atrophy in the retinal layer with thin photoreceptor cells, bipolar cell layers, and ganglion cell layers in the late stage (**Figure 2o**, blue arrow) and abnormal proliferative structures in the subretinal space (**Figure 2o**, circle). The histopathological damages in all dead animals were observed as early as day 7 postimmunization, with an average grade of 2–3. Histopathological scores of the inflammatory peak in 50-μg-induced EAU with an average grade of 1 were lower than those in 300-μg–induced and 800-μg–induced EAU with IRBP_1197-1211_ and in 800-μg–induced EAU with BR14 (**Figure S1**).

### Infiltration of Innate Immune Cells and Adaptive Immune Cells Into the Eyes of Tree Shrews With EAU

**Figure 3:**
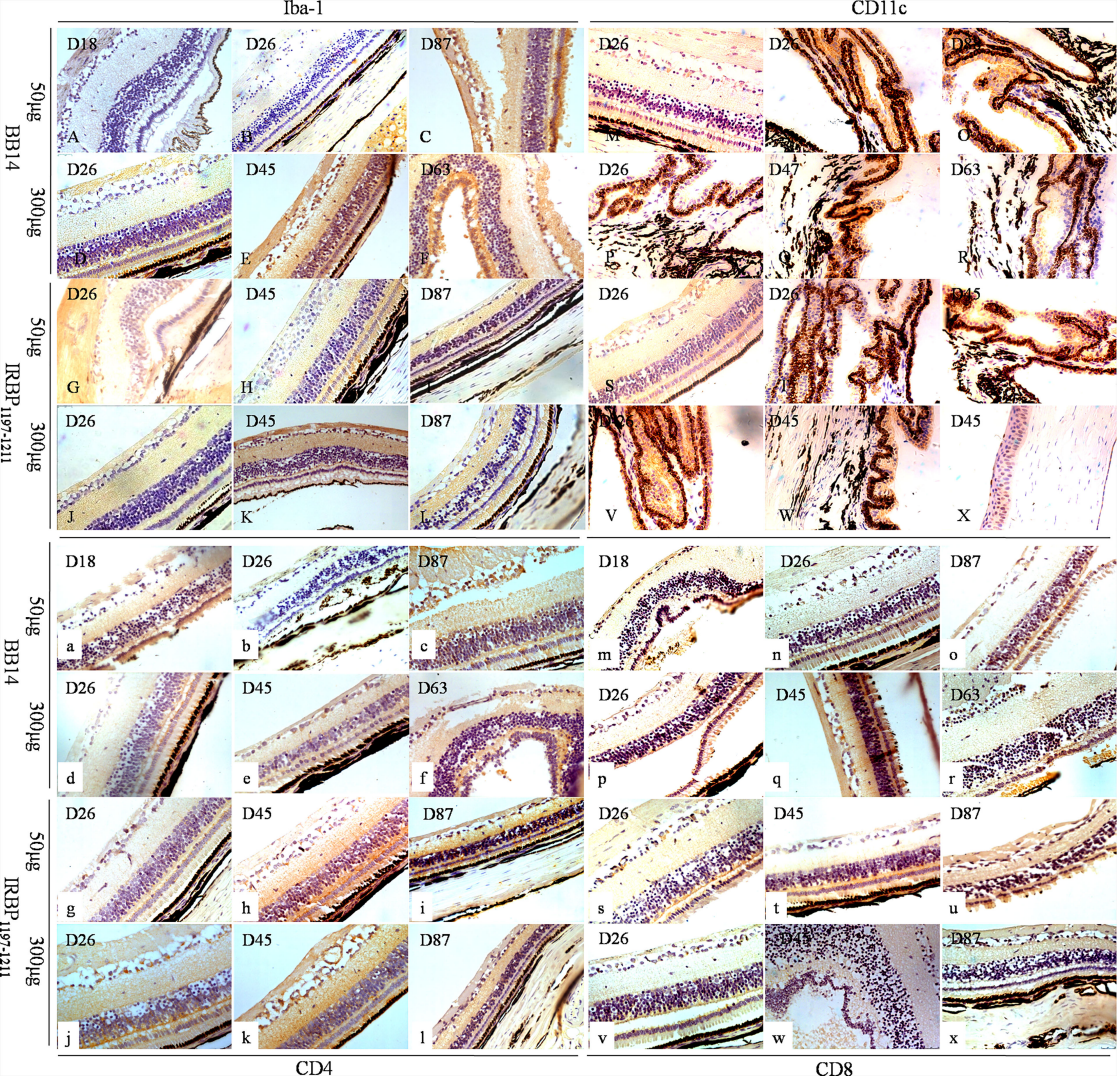
Immune phenotypes of EAU in tree shrews immunized with BR14 or IRBP_1197–1211_ and human uveitis. Iba-1 was mostly expressed on the wing cells and basal cells of the cornea; on the non-pigmented epithelium of the ciliary processes; on the choroid layer; on the visual cell layer, inner nuclear layer, and ganglion cell layer **(A–L)**. CD11c was predominately expressed on the non-pigmented epithelium of the ciliary processes and sparsely expressed on the photoreceptor cell layer, ganglion cell layer, and corneal epithelium layer **(M–X)**. Positive expression of CD4 was observed in the cornea and various layers of the retina **(a–l)**. Weak and sparse expression of CD8 was found in the cornea and various layers of the retina **(m–x)**.

**Figure 4:**
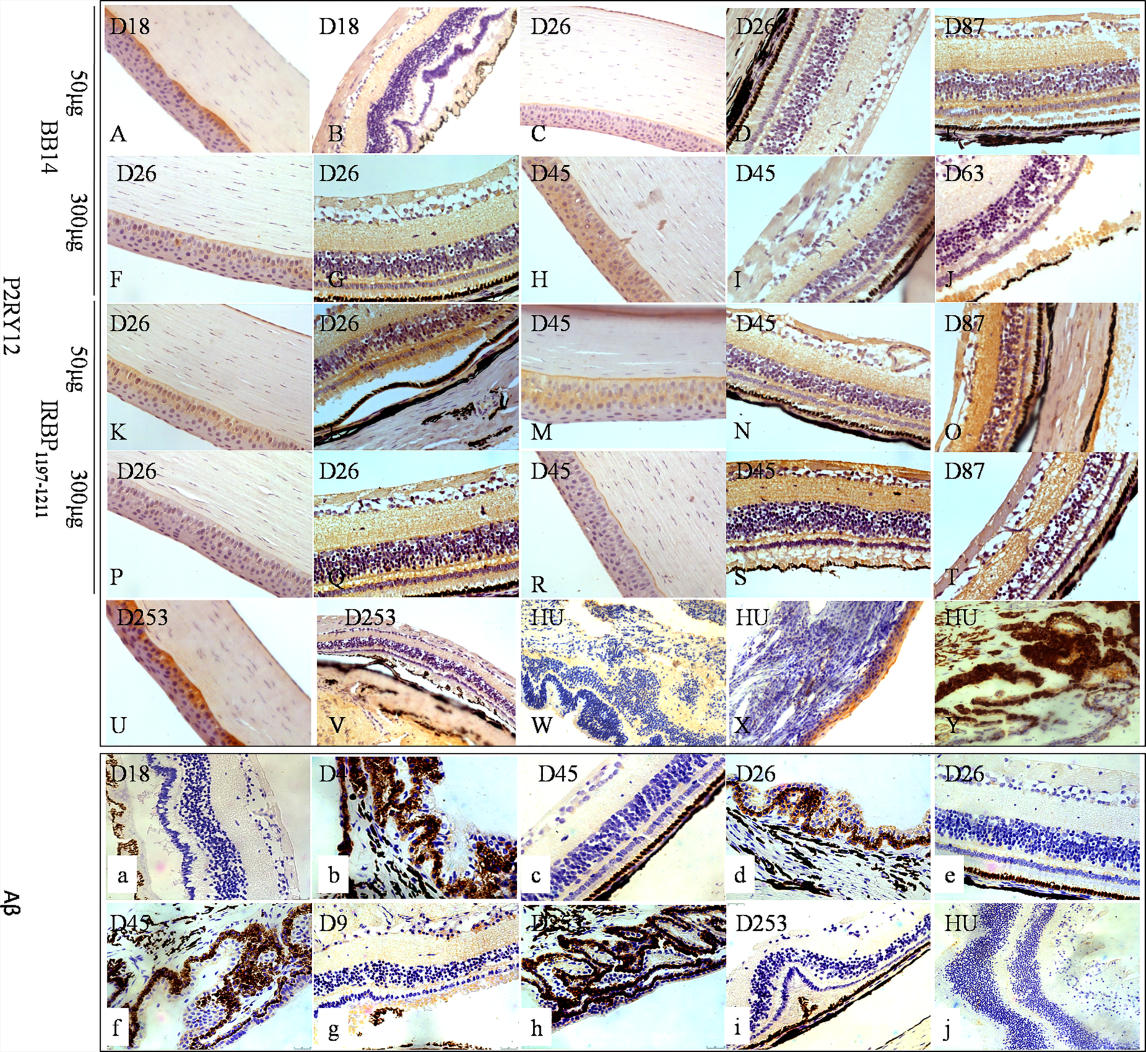
The expressions of P2RY12 and Aβ of human uveitis and tree shrew EAU. P2RY12 **(A–Y)** was expressed on the cornea, but not on the retina, on day 18 postimmunization with 50 µg of BR14 **(A, B)** and day 235 postimmunization with 800 µg of IRBP **(U–W)**. On day 26 postimmunization with 50 µg of BR14, P2RY12 was weakly expressed on the cornea and retina **(C, D)**. However, strong expression of P2RY12 was observed on the cornea and various layers of the retina on day 87 postimmunization with 50 µg of BR14 and days 26, 45, 87, and 235 postimmunization with 50 µg of BR14 **(C, D)**, 300 µg of BR14 **(F–J)**, 50 µg of IRBP **(K–O)**, 300 µg of IRBP **(P–T)**, 800 µg of IRBP **(U, V)**, and human uveitis **(W–Y)**. The positive expression of Aβ **(a-j)** was not observed in the retinal lesions on day 18 postimmunization with 50 µg of BR14 **(a)**, but weak expression was observed in the ciliary processes, retinal layer, and retinal lesions on day 45 postimmunization with 50 µg of BR14 animals **(b, c)**, on days 26 **(d, e)** and 45 **(f)** post-immunization with 300 µg of BR14, on days 9 **(g)** and 235 **(h, i)** postimmunization with 800 µg of IRBP, and in the human retina **(j)**.

At the peak of disease (days 25–45) induced by 50 µg of BR14, 300 µg of R14, 50 µg of IRBP_1197–1211_, and 300 µg of IRBP_1197–1211_, CD45 cells were observed in the choroid and the retina; notably, these cells included CD11c^+^ dendritic cells (DCs), CD68^+^/ionized calcium–binding adaptor molecule-1 (Iba-1)^+^ macrophages, purinergic receptor P2Y, G-protein–coupled 12 (P2RY12)^+^ microglia, CD4^+^ T-cells, and CD8^+^ T-cells (**Figures 3**, **4**). Moreover, periodic acid–Schiff (PAS) staining and oil red O staining yielded negative results for subretinal amorphous eosinophilic deposits, but the results were partly positive following staining by Iba-1 (**Figures 3C, F**), P2RY12 (**Figures 4E, J, S**), CD8 (**Figures 3r, t, w**), and amyloid β-protein (Aβ) (**Figures 4g, i**) at the site of damage of the retina at the peak or late stages of disease.

### Tree Shrews With EAU Show Antigen-Specific T-Cell Proliferation After EAU Induction with IRBP_1197–1211_ or BR14

**Figure 5:**
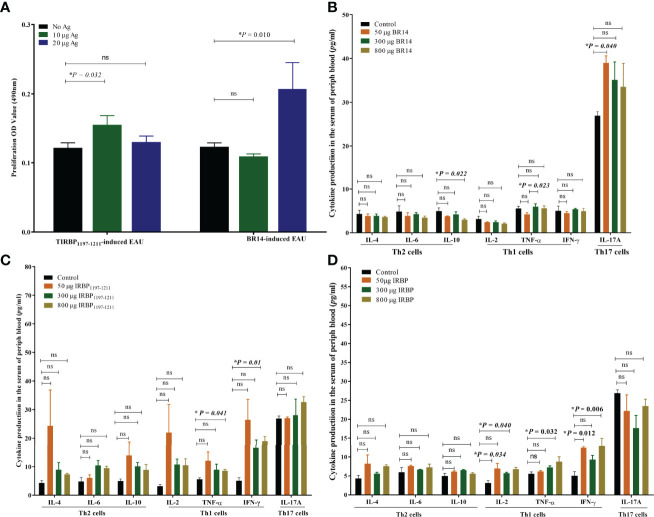
Tree shrews with EAU showed a systemic IRBP-specific immune response *in vivo* and *in vitro*. **(A)** Lymphocytes from the spleen of EAU tree shrews induced with 800-µg BR14 and 800-µg IRBP_1197–1211_ were collected on day 25 post immunization and were stimulated with or without the BR14 and IRBP peptides. The proliferative response was measured *via* the MTT assay. The affected tree shrew showed the Ag-specific proliferative response. Data are expressed as mean ± SEM and are representative of two independent experiments. *P < 0.05 in one-way ANOVA. Three tree shrews were used in each group. **(B)** indicates that decreased IL-10 levels in the 800-μg BR14–induced group and increased IL-17 levels in the 50-μg BR14–induced group on day 25. **(C)** indicates an increased level of Th1 cytokine TNF-α and IFN-γ in the 800-μg IRBP–induced group on day 25. **(D)** shows increased IL-2 and IFN-γ levels in the 50-μg IRBP–induced group and increased IL-2, IFN-γ, and TNF-α levels in the 800-μg IRBP-induced group on day 40. Data are expressed as mean ± SEM. *P < 0.05 in one-way ANOVA. ns, no significant difference. Three tree shrews were used in each group.

The antigen-presenting cells (APCs) and T-cells of spleens from tree shrews with EAU as well as from healthy controls were collected on day 25 postimmunization with 800 μg of antigens and were *in vitro* co-cultured with or without antigen. The T-cell proliferation assay was determined by MTT stimulated by APCs with or without antigen for 72 h. We observed a significantly higher proliferation of T-cells in the tree shrew EAU group immunized with 800 μg of IRBP in the presence of 10 μg of IRBP and immunized with 800 μg of BR14 in the presence of 20 μg of BR14 compared to with no antigen stimulation, respectively (**Figure 5A**).

### Th1 or Th17 Cytokine Production of Peripheral Blood in Tree Shrews With EAU

To evaluate cytokine production of the peripheral blood of tree shrews with EAU, serum was obtained from the peripheral blood of the control healthy tree shrews and tree shrews with EAU induced with 50 μg of BR14, 300 μg of BR14, 800 μg of BR14, 50 μg of IRBP, 300 μg of IRBP, or 800 μg of IRBP by centrifugation. The Th17 signature cytokine interleukin (IL)-17A; Th1 signature cytokines IL-2, interferon (IFN)-γ, and tumor necrosis factor alpha (TNF-α); Th2 signature cytokines IL-4, IL-6, and IL-10 of serum in the tree shrews immunized with R14 and IRBP_1197–1211_ on day 25 and day 40 post immunization were detected using cytometric bead array. Regarding the protective Th2 cytokine level, the FACS results showed a decreased IL-10 level in the 800-μg BR14–induced group on day 25 compared to the healthy control group. For proinflammatory cytokine production, the 800-μg IRBP–induced group on day 25 showed increased levels of Th1 cytokine TNF-α and IFN-γ, and on day 40 the 50-μg IRBP–induced group and the 800-μg IRBP–induced group showed increased IL-2, IFN-γ, and TNF-α levels compared to the healthy control group on day 40. Meanwhile, an increased Th17 cytokine IL-17A level on day 25 was observed in the 50-μg BR14–induced group compared to the control group (**Figures 5B–D**).

### Enrichment of DEGs in Tree Shrews With EAU Occurred Mostly in Adenylate Cyclase Activity, Ruffle, and Cell Development

**Figure 6:**
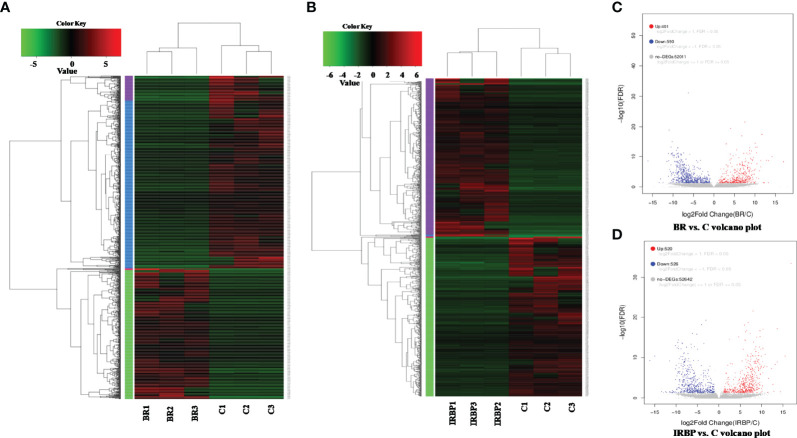
Heatmap and volcano plot of the DEGs in different samples. The heatmap indicates that the expression patterns were significantly different in the eyes of BR14-induced animals **(A)** and IRBP-induced animals **(B)** compared to those of the control group. BR: Eyes in the BR14-induced group. IRBP: Eyes in the IRBP-induced group. C: Eyes in the healthy control group. **(C)** A volcano plot of the DEGs shows 401 upregulated genes (red dots) and 590 downregulated genes (blue dots) in the eyes of the BR14–induced group compared to the control group. **(D)** A volcano plot of the DEGs shows 520 upregulated genes (red dots) and 526 downregulated genes (blue dots) in the eyes of the IRBP-induced group compared to the control group.

**Figure 7:**
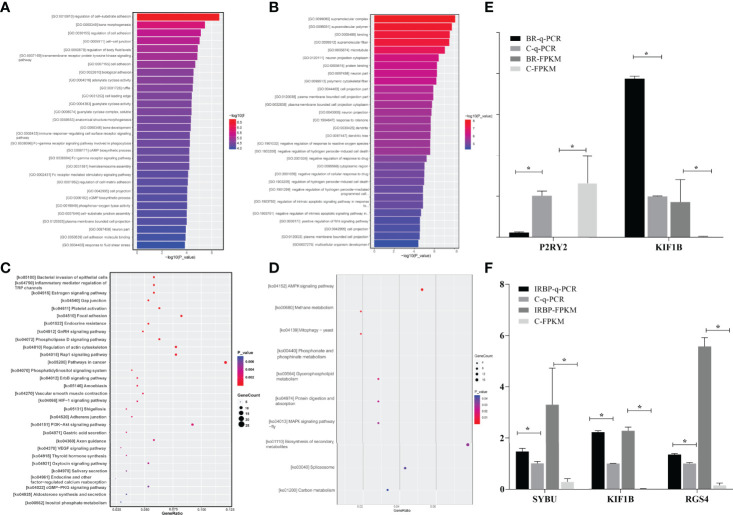
The enrichments of the DEGs and validation of the DEGs in RNA-seq by qPCR assay. **(A)** indicates that GO of the 991 DEGs in the BR14-induced group is involved in 20 biological processes, 13 cellular components, and 9 molecular functions. **(B)** indicates that GO of the 1046 DEGs in the IRBP-induced group is involved in 22 biological processes, 16 cellular components, and 8 molecular functions. The lower and upper abscissae show the numbers and ratios of annotated DEGs, respectively. The ordinate shows the categories of differential expression genes according to biological process, cellular components, and molecular function. **(C)** indicates that the DEGs in the BR14-induced group were enriched in 5 human diseases, 4 cellular processes, and 11 organismal systems by enrichment analysis of the KEGG pathway. **(D)** indicates that the DEGs were enriched in 5 human diseases, 4 cellular processes, and 11 organismal systems by enrichment analysis of the KEGG pathway. The abscissa represents the name and class of the KEGG pathway enrichment of the DEGs. The ordinate represents the enrichment of the ratio calculated by the formula of enrichment ratio = sample number/background number). **(E)** shows the expression level of DEGs by qPCR. The mRNA levels were expressed as the mean ± SEM of ΔCt in three biological replicates. *P < 0.05 in t-test. **(F)** shows the expressions of DEGs by RNA-Seq. Data are expressed as the mean ± SEM of FPKM in three samples. *P < 0.05 in t-test.

To understand what differences existed between the eyes of tree shrews with and without EAU, DEG analysis was performed using RNA-seq. After the qualified control evaluation, the transcript expression was obtained based on FPKM. The clusters of DEGs in eye samples from tree shrews immunized with 800-µg BR14 or 800-µg IRBP_1197–1211_ and control healthy tree shrews showed that there was a high correlative index between the samples within the same group; however, there was a low correlative index between the samples of different groups. The heatmap revealed that the expression patterns were significantly different in the eyes of tree shrews with EAU compared to those of the control group (**Figures 6A, B**). A total of 991 DEG transcripts were identified (**Figure 6C**), and the upregulated and downregulated transcripts totaled 401 and 590, respectively, in the eyes of the BR14-induced tree shrews compared to those of the control group. The enriched Gene Ontology (GO) categories in the BR14-induced group were spread across 50 biological processes, 9 cellular components, and 11 molecular functions; the top 30 enriched GO categories are mostly involved in the regulation of cell–substrate adhesion, bone morphogenesis, and the transmembrane receptor protein tyrosine kinase signaling pathway (**Figure 7A**), which enabled us to further explore their potential role in EAU. Meanwhile, a total of 1,046 DEG transcripts were identified (**Figure 6D**), and the upregulated and downregulated transcripts totaled 520 and 526, respectively, in the eyes of the IRBP-induced tree shrews compared to those of the control group. Here, the enriched GO categories were spread across 42 biological processes, 25 cellular components, and 11 molecular functions, and the top 30 GO categories of DEGs in IRBP_1197–1211_-induced eyes are involved in the supramolecular complex, supramolecular polymer binding, supramolecular fiber, and microtubule (**Figure 7B**).

As shown in the Kyoto Encyclopedia of Genes and Genomes (KEGG) enrichment pathway of the messenger RNAs (mRNAs) (**Figure 7C** and **Table S1**), five human diseases, including bacterial invasion of epithelial cells, endocrine resistance, amoebiasis, and shigellosis; eight environmental information processing; four cellular processes including inflammatory mediator regulation of TRP channels; 11 organismal systems were significantly enriched in the eye group of BR14-induced tree shrews. Especially, the crucial DEGs, *including P2RY2, adenylate cyclase 4 (ADCY4), ADCY6, and recombinant nitric oxide synthase 3 (NOS3)* were involved in most enrichment pathways in the BR14 group (**Table S2**). As shown in the KEGG enrichment pathway of the mRNAs (**Figure 7D** and **Table S3**), two environmental information processes associated with the AMP-activated protein kinase (AMPK) signaling pathway and MAPK signaling pathway and five metabolisms were slightly enriched in the eye group of IRBP-induced tree shrews. Of note, RGS4 related to GTPase activity of G-protein alpha subunits was significantly upregulated in the IRBP_1197–1211_ group.

### Verification of DEGs by qPCR

The expression levels of four DEGs were detected by qPCR to determine the content of the DEG library. **Figures 7E, F** show the same expression patterns in the qPCR and RNA-seq analyses, thereby validating our results of RNA-seq.

### Treatment of EAU Induction by IRBP_1197–1211_ With an RGS4 Inhibitor and DHA Significantly Ameliorates the EAU

**Figure 8:**
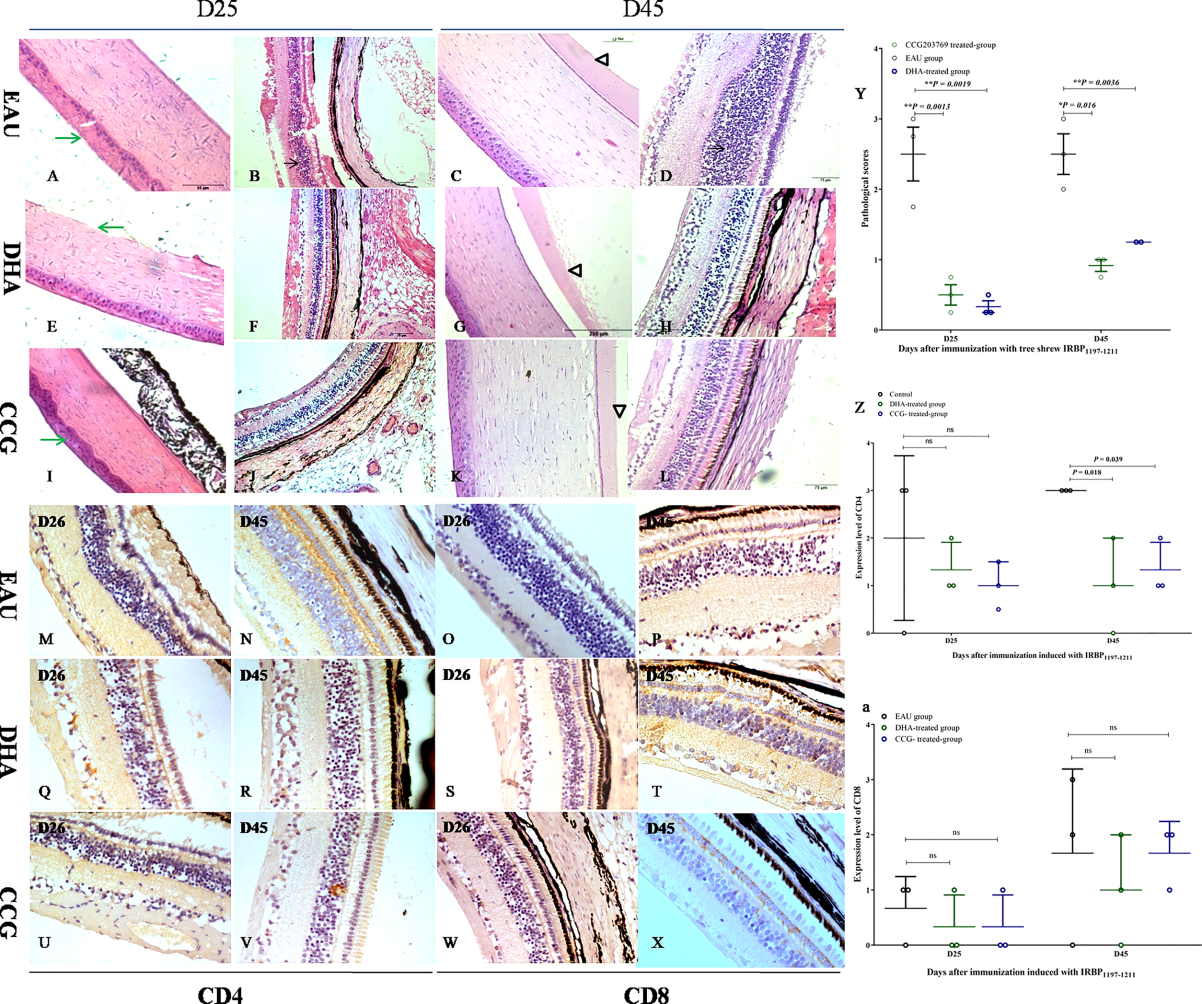
RGS4 inhibition and DHA significantly ameliorated EAU. The immunized tree shrews treated with both CCG 203769 and DHA developed significantly milder ocular inflammation (CCG 203769-treated group: **(A–D)**; DHA-treated group: **(E–H)**; oil-treated group: **(I–L)** and achieved a marked reduction in the EAU histopathological score **(Y)** than the corn oil-treated immunized tree shrews on days 25 and 45 postimmunization. The expression of CD4 T-cells in CCG 203769 and DHA-treated groups on day 45 was significantly reduced compared with the control-treated tree shrews **(M, N, Q, R, U, V, Z)**. However, the expression of CD8 T-cells was not significantly different between the CCG 203769-treated tree shrews **(W–X)**, the DHA-treated tree shrews **(S–T)**, and the control-treated tree shrews **(O–P, a)**. ns, no significant difference. *P < 0.05. **P < 0.01.

We then evaluated whether a DEG RGS4 inhibitor and DHA would inhibit the development of EAU. As the average EAU scores induced in 50-μg-Ag–induced and 300-μg-Ag–induced tree shrews were lower than that of the 800-μg-Ag–induced EAU group, we chose the 800-μg-IRBP–induced EAU tree shrews, which developed stronger pathological lesions using the same experimental protocol for EAU induction. As shown in **Figure 8**, the immunized tree shrews treated with both CCG 203769 and DHA achieved a marked reduction in EAU histopathological scores (**Figure 8Y**) and developed significantly milder ocular inflammation than the corn oil-treated immunized tree shrews (**Figures 8A–L**). As shown in **Figures 8M–X**, the expression of CD4^+^ T-cells of the CCG 203769-treated tree shrews and the DHA-treated tree shrews was significantly reduced on day 45 compared with the control-treated tree shrews (**Figure 8Z**). However, the expression of CD8^+^ T-cells was not significantly different between the CCG 203769–treated tree shrews, the DHA-treated tree shrews, and the control-treated tree shrews (**Figure 8a**). Further cytokine detection show no significant change in Th2 signature cytokines, Th1 signature cytokines, and Th17 signature cytokine after treatment with CCG 203769 and DHA on day 45 (**Figure S2**).

## Discussion

We investigated the development, characteristics, and treatment of a novel EAU model enacted in tree shrews. The results indicated that bovine R14 and tree shrew IRBP_1197–1211_ could induce chronic inflammation in the retina and uvea with the typical characteristics seen in humans and mice. The novel tree shrew EAU model animals showed inflammatory infiltration of eyeball layers, retinal folding, multiple innate immune cells (retinal microglia, macrophages, and dendritic cells), and adaptive immune cells (CD4^+^ T-cells and CD8^+^ T-cells), a significantly enriched neuron part in the GO function analysis, and bacteria invasion of epithelial cells in BR14-immunized eyes and AMPK signaling pathway in the IRBP_1197–1211_-immunized eyes. Moreover, the DEG RGS4 inhibitor CCG 203769 could alleviate the severity of EAU by reducing the expression of CD4 T-cells on day 45 postimmunization in the 800-μg-IRBP–induced tree shrew eyes.

In humans, uveitis presents multiple distinct clinical findings, such as red and painful eyes and conjunctival injection (20). In the present study, in line with the variable clinical features in human uveitis, conjunctival hyperemia, injection of the cornea, and retinal degeneration were present at various stages in the development of tree shrew EAU. Histopathological results confirmed diffuse inflammatory infiltration of the cornea, uveal, and retina; disorganization in the retinal pigment epithelium and the inner nuclear layer, and retinal folding. Those pathological features in our study can be supported by data available from studies on classical B10RIII mice (11, 12), B10.A mice, C57BL/6J mice with EAU (21), and human uveitis (20). Those results suggest that the tree shrew EAU model in the present study can represent human uveitis and previously reported classical EAU in mice.

However, the unique features of outer segment loss or amorphous eosinophilic deposits in the inner segment of photoreceptors in the present study are distinguished from the uveitis in C57BL/6J and B10RIII mice and Lewis rats. In human and nocturnal rodents, cones constitute only a small fraction of the total number of photoreceptors, whereas, in tree shrews, cones account for 95% of the retina and rods account for 5%. Ingram and colleagues demonstrated that cones have a higher ATP expenditure than rods and thereby provide the reasons why cones in the diseased retina first lose their outer segments (22–24). In addition, the outer segment requires a tremendous amount of energy to sustain the function and structural renewal for normal homeostasis. Thus, it is not surprising that the stressors elicited by retinal antigens can further elevate metabolic demand and thereby lead to damage of the outer segment in the present study. In addition, the degeneration of their support cells, such as pigment epithelial cells, can also explain the photoreceptor dysfunction and death seen in the present study. Our observations may provide some explanation for why patients with uveitis suffer high consequences in the fovea with clustered cones when glucose transport or availability is diminished owing to the high energy requirements.

Microglia is another type of support cell—a specialized kind of macrophage-derived from yolk sacs—essential for the maintenance of photoreceptor health and function and is also the primary effector of the retinal inflammatory response to acute and chronic disorders (25). It has been demonstrated that microglia initiate the infiltration of immune cells into the retina during the early stages of EAU, and their depletion ablates the access of circulating immune cells into the retina (26). In our present study, P2RY12^+^ microglia were observed at the lesions of the inner segment of photoreceptor cells and the ganglion cell layer on day 9 postimmunization with 800 μg of IRBP_1197–1211_ in the tree shrew eye, suggesting that microglia are involved in the development of disease in tree shrews during the early phase of EAU. Previous studies have also indicated that activated microglia can interact with antigen-specific T-cells and with MHC class II^+^ cells and CD11b^+^ APC cells, and they further trigger and amplify the retinal inflammation in EAU. Indeed, our immunohistochemical results further indicated that CD11c^+^ dendritic APCs, Iba-1^+^ macrophages, P2RY12 +^+^ microglia, CD4^+^ T-cells, and CD8^+^ T-cells present at the photoreceptor cell layer, inner nuclei layer, and ganglion cell layer in the peak and late phases of EAU. These results suggest that CD11c^+^ APC cells, Iba-1^+^ macrophages, microglia, CD4^+^ T-cells, and CD8^+^ T-cells are the predominant cells infiltrating into the retina and uvea. Of interest, cell debris of the inner segment at the lesions of photoreceptor cells layers positively expressed Iba-1, P2RY12, CD8, and Aβ protein but was negatively stained by PAS staining and Oil O staining, suggesting that macrophages/microglia and CD8 T-cells may be involved in cell debris formation by removing the photoreceptor cells damaged in response to the inflammatory stimulus. It has been demonstrated that the core marker P2RY12 of microglia is highly expressed on quiescent and activated non-inflammatory M2 microglia (27, 28). Under pathological conditions, microglial P2RY12 could trigger microglia motility and migration from the outer plexiform layer, inner plexiform layer, and ganglion cell layer of the retina toward the lesion site while responding to ATP release from damaged cells (29). During this chemotaxis course, microglia adopt an amoeboid morphology, exert phagocytosis to clear the infected cells or cellular debris, and repair the damaged tissue from a ramified morphology (29).

It has been reported that elevated levels of Th1 cytokines (IFN-γ, TNF-α, and IL-2) were detected in the serum of Behçet’s patients (30). In alignment with Behçet’s patient cytokine profile, elevated levels of proinflammatory Th1 cytokines in the serum of 50-μg IRBP_1197–1211_ and 800-μg- IRBP_1197–1211_-induced tree shrews on days 25 and 40 were shown in our present study, suggesting a pathogenic Th1 response on day 25. However, in BR14-induced EAU, the findings of an increased Th17 cytokine level in low BR14-induced EAU and a decreased Th2 cytokine level in high BR14-induced EAU demonstrated that BR14-induced EAU in tree shrews is characterized by pathogenic Th17 responses or protective Th2 responses. In accordance with the findings in the present study, IL-17A is an important pathogenic cytokine in uveitis and EAU (2, 31).The increased TNF-α level in the serum of 800-μg- IRBP_1197–1211_-induced tree shrews on days 25 and 40 was observed, suggesting that combinations of multiple Th1 cytokines may be involved in the severity of pathological lesions in high IRBP_1197–1211_-induced EAU.

The retina is primary targets of inflammatory cells attacking the development of autoimmune uveitis. In particular, the typically damaged tissues or cells in human uveitis and animal EAU are predominantly involved in the photoreceptor cell layer, blood vessels, and optic discs. It is known that photoreceptors are highly specialized neurons that contain rod and cone photoreceptors in the retina. Cones and rods morphologically are comprised of four subcellular compartments: an outer segment, inner segment, somata, and synaptic terminal. The photoreceptor outer segment is a modified sensory cilium containing microtubular axoneme and hundreds of flattened disc-shaped membranes that provide vast light-absorbing surfaces for phototransduction (32), whereas the inner segment houses the mitochondria, which provide ATP to the outer segment and support the Na^+^/K^+^ ATPase located in the inner segment plasma membrane (33), endoplasmic reticulum, and Golgi apparatus to meet the energy and biosynthetic needs of cells. Human and animal research has indicated that uveitis is attributed to photoreceptor damages, which predominately cause the vision loss reported in affected patients or animals. In line with previously reported studies, photoreceptor disorganization, degeneration, necrosis, and loss were observed on days 25–235 postimmunization with BR14 and IRBP_1197–1211_ peptides in the present study. Of interest, the adenylate cyclase activity was significantly enriched in the eyes of BR14-induced tree shrews compared to healthy eyes. The upregulated genes, such as *natriuretic peptide receptor (NPR)2, ADCY1, ADCY4, ADCY6, ADCY9, and LOC102483497* related to adenylate cyclase and guanylate cyclase activities in the DEGs may explain the damage mechanism of the light-sensitive photoreceptor outer segment, where phototransduction occurs, and the metabolic-related inner segment during the development of uveitis in tree shrews. In alignment with the present results, Rao and colleagues also reported a decreased expression of ATP synthase in the early EAU retina (34). Therefore, we propose that those upregulated genes may cause abnormal function of the photoreceptor layer and thus may explain the mechanism of photoreceptor damage in uveitis in tree shrews.

In particular, the gene *RGS4*, which can limit heterotrimeric G protein activity and promote Gα inactivation related to the regulation of the G-protein signaling domain, were significantly increased in the eyes of IRBP_1197–1211_–induced tree shrews compared to control eyes, suggesting that RGS4 is involved in the pathogenesis of tree shrew EAU in the current study. It has been demonstrated that RGS4 plays a significant role in various diseases, such as asthma (35), epilepsy (36), and Parkinson’s disease (37), and inhibition of RGS4 with their antagonists can alleviate these diseases, suggesting that RGS proteins might be the potential therapeutic target in those conditions. Based on these facts, we further evaluated the intervention effect of the RGS antagonist CCG 203769 on the development of EAU in tree shrews, and our treatment results demonstrated that inhibition of RGS4 with antagonist CCG can suppress the severity of inflammation by reducing the expression of CD4 cells. The cytokine results show no significant change in Th2 signature cytokine, Th1 signature cytokines, and Th17 signature cytokines after treatment with CCG 203769 and DHA, suggesting that other CD4-T subtype responses may be involved in the disruptive effect. Although the details of the mechanism by which RGS4 contributes to uveitis await further study in the future, the findings in our study and previous reports support the notion that RGS4 may be a potential therapeutic target in uveitis and other diseases.

**Figure 9:**
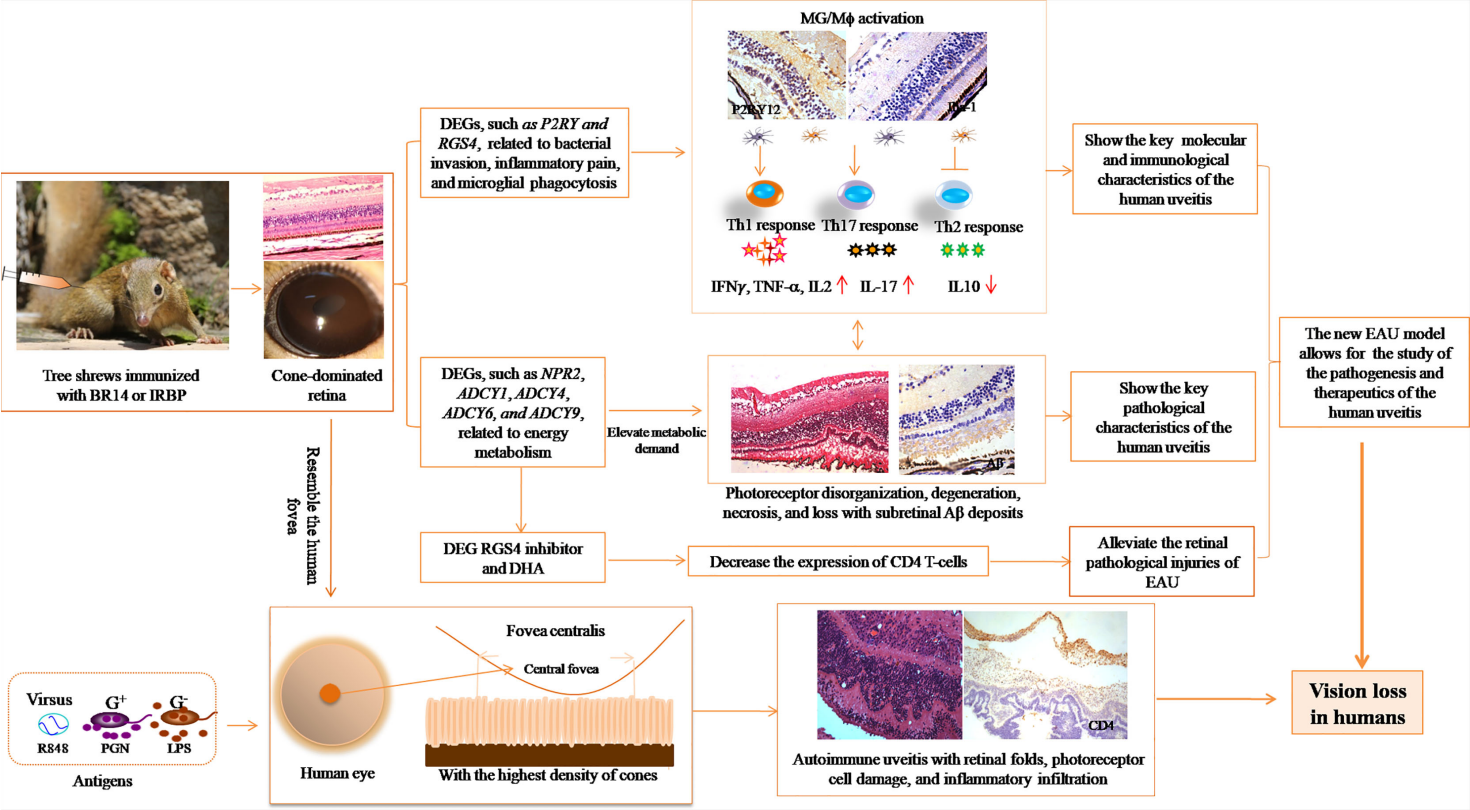
Illustration of the significance of the new EAU model in tree shrews for studying autoimmune uveitis.

In conclusion, the described tree shrew uveitis model has the potential to be a less costly platform to study the pathogenesis of and therapeutics for fovea-involved visual disturbance after inflammation in human uveitis (**Figure 9**).

## Data Availability Statement

The original contributions presented in the study are publicly available. This data can be found here: https://www.ncbi.nlm.nih.gov/bioproject/PRJNA797233.

## Ethics Statement

The study was reviewed and approved by the Ethics Committee of Chongqing Medical University.

## Author Contributions

FC and LL designed and performed the experiments, performed the data analysis, and wrote the manuscript. KH, HH, GY, JG, FZ, and YM performed the experiments. KH, HH, and JL were involved in data analysis, and wrote the manuscript. JL, YY, WZ, YC, and NZ participated in the experiments. All of the authors have given their permission to be named. All authors contributed to the article and approved the submitted version.

## Funding

The authors are grateful for the support from the National Natural Science Foundation Project (Grant no. 81670843) and the Project of Yuzhong District Science and Technology Bureau (Grant no. 20200127).

## Conflict of Interest

The authors declare that the research was conducted in the absence of any commercial or financial relationships that could be construed as a potential conflict of interest.

## Publisher’s Note

All claims expressed in this article are solely those of the authors and do not necessarily represent those of their affiliated organizations, or those of the publisher, the editors and the reviewers. Any product that may be evaluated in this article, or claim that may be made by its manufacturer, is not guaranteed or endorsed by the publisher.
