# Supplementary material for: A Novel Tree Shrew Model of Chronic Experimental Autoimmune Uveitis and Its Disruptive Application
Source: Front Immunol. 2022 May 27;13:889596. doi: 10.3389/fimmu.2022.889596 (PMC9196886; doi:10.3389/fimmu.2022.889596)
Supplement: Supplementary file 1 [file DataSheet_1.pdf]

Supplementary Material

Supplementary Tables

TABLE S1. Primers used for verification of DEGs by q-PCR

Gene	sequence	PCR Products (bp)
Actin	Forward sequence (5'-3'): CCCAGTCCTCACCCAAATACA	183
	Reverse sequence (5'-3'): GGGAGACCATAAGCCTTCATAC	
RGS4	Forward sequence (5'-3'): GCTGCGAAACGAAGACACC	139
	Reverse sequence (5'-3'): ACGGAAAGGGATGCTGATG	
KIF1B	Forward sequence (5'-3'): GTAGCCCCTGCCGGAATT	198
	Reverse sequence (5'-3'): CGAGACACGCTCAGCACTCT	
P2RY2	Forward sequence (5'-3'): TACTACTCCTTCCGCTCACTCA	103
	Reverse sequence (5'-3'): CCAGGCAACTGTTGGCACT	
SYBU	Forward sequence (5'-3'): AGGAGGTCACGGAGGAGGG	139
	Reverse sequence (5'-3'): CCAGGGGTGGAATGAACAAA	

TABLE S2. The pathway enrichment of differently expressed genes (DEGs) in the BR14 group

Description	Category	P-adjust	Gene name
Bacterial invasion of epithelial cells	Human Diseases	0.000265	XLOC_020866, MAPK15, XLOC_048568, XLOC_050538, XLOC_050836, XPNPEP1, PDLIM4, RBM26, SEPT9, ELMO2, DOCK1, CAV1, SRC
Inflammatory mediator regulation of TRP channels	Organismal Systems	0.000572	XLOC_003642, STX16, SNTA1, PPP6R2, XLOC_050538, LOC102483497, P2RY2, ADCY6, ITPR3, PRKCD, SRC, ADCY4
Estrogen signaling pathway	Organismal Systems	0.000572	XLOC_003642, STX16, GADD45G, XLOC_050538, FRMD4B, LOC102483497, ATF6B, ADCY6, GABBR1, ITPR3, ATF6B, PRKCD, SRC, ADCY4, NOS3
Gap junction	Cellular Processes	0.000951	XLOC_003642, STX16, ZGRF1, LOC102483497, TRPM1, ADCY6, LOC102496850, ITPR3, SRC, ADCY4, MAP2K5
Platelet activation	Organismal Systems	0.001556	XLOC_003642, STX16, PPP6R2, XLOC_050538, XLOC_050836, LOC102483497, ARHGEF1, COL1A1, CYTH3, ITPR3, SRC, ADCY4, NOS3
Focal adhesion	Cellular Processes	0.002684	XLOC_020866, MAPK15, XLOC_048568, XLOC_050538, XLOC_050836, XLOC_061360, XLOC_061360, NA, ABCA4, CRYBA2, RBM26, LAMB3, COL1A1, DOCK1, CAV1,

			SRC, FLT4, PAK6
Endocrine resistance	Human Diseases	0.004674	XLOC_003642, STX16, MAPK15, PPP6R2, XLOC_050538, LOC106736342, LOC102483497, ADCY6, SRC, ADCY4, MDM2
GnRH signaling pathway	Organismal Systems	0.004674	XLOC_003642, STX16, PPP6R2, LOC102483497, ADCY6, PRKCD, ITPR3, SRC, ADCY4, MAP2K4
Phospholipase D signaling pathway	Environmental Information Processing	0.004674	XLOC_003642, STX16, XLOC_012724, SNTA1, XLOC_050538, XPNPEP1, ZGRF1, LOC102483497, RALGDS, RALGDS, CYTH3, ADCY6, RAPGEF3, CYTH3, ADCY4
Regulation of actin cytoskeleton	Cellular Processes	0.006887	ATP5ME, XLOC_020866, MAPK15, XLOC_036533, XLOC_048568, XLOC_050538, XLOC_050836, CDC16, XLOC_061360, ZGRF1, RBM26, XLOC_086978, ARHGEF1, ACTN1, DOCK1, SRC, PAK6
Rap1 signaling pathway	Environmental Information Processing	0.008449	XLOC_003642, STX16, ACO1, SNTA1, PPP6R2, XLOC_050538, XLOC_050836, ZGRF1, LOC102483497, XLOC_086978, RALGDS, RALGDS, ADCY6, RAPGEF3, SRC, ADCY4, FLT4
Pathways in cancer	Human Diseases	0.011204	XLOC_003642, HERC1, STX16, NXPE3, SNTA1, MAPK15, XLOC_048568, XLOC_050538, XLOC_050836, ZGRF1, ABCA4, LOC102483497, XLOC_086978, EGLN2, ARHGEF1, MECOM, SPI1, LAMB3, RALGDS, RALGDS, ABL1, GNGT2, ADCY6, ADCY4, AXIN2, MDM2
Phosphatidylinositol signaling system	Environmental Information Processing	0.011361	XLOC_012724, SNTA1, OSBP2, XLOC_050538, PTPRM, INPP4A, CDIPT, ITPR3
ErbB signaling pathway	Environmental Information Processing	0.021818	SNTA1, MAPK15, XLOC_050538, XLOC_061360, ZGRF1, ABL1, SRC, PAK6, MAP2K4
Amoebiasis	Human Diseases	0.021818	STX16, MAPK15, XLOC_048568, XLOC_050538, LOC102483497, RBM26, ACTN1, LAMB3, COL1A1
Vascular smooth muscle contraction	Organismal Systems	0.025525	XLOC_003642, STX16, LOC102483497, ARHGEF1, COL1A1, ADCY6, ITPR3, SRC, ADCY4, NOS3
HIF-1 signaling pathway	Environmental	0.027712	ACO1, SNTA1, CHMP6, XLOC_050538, ZGRF1, LSAMP, EGLN2, SERPINE1, NOS3

	Information Processing		
Shigellosis	Human Diseases	0.027712	XLOC_020866, PPP6R2, XLOC_050836, RBM26, ELMO2, ABL1, DOCK1, SRC
Adherens junction	Cellular Processes	0.030368	XLOC_036533, BCAN, AGRN, TRPM1, RBM26, XLOC_086978, ACTN1, SRC
PI3K-Akt signaling pathway	Environmental Information Processing	0.033711	MAPK15, HECTD1, GADD45G, XLOC_048568, XLOC_050538, XLOC_050836, STX10, NA, CRYBA2, XLOC_086978, SGK1, ATF6B, LAMB3, GNGT2, COL1A1, ATF6B, MDM2, TEDC2, ACO1, FLT4, NOS3, ZGRF1
Gastric acid secretion	Organismal Systems	0.034481	XLOC_003642, STX16, GALNT11, LOC102483497, ADCY6, ITPR3, ADCY4
Axon guidance	Organismal Systems	0.034511	HEPACAM, SNTA1, MAPK15, PPP6R2, XLOC_050538, XLOC_050836, LOC102492209, XLOC_061360, NDST4, ABL1, SRC, PAK6
VEGF signaling pathway	Environmental Information Processing	0.042375	SNTA1, MAPK15, PPP6R2, XLOC_050538, SRC, NOS3
Thyroid hormone synthesis	Organismal Systems	0.042375	XLOC_003642, STX16, GADD45G, LOC102483497, ATF6B, ADCY6, ITPR3, ADCY4
Oxytocin signaling pathway	Organismal Systems	0.042375	XLOC_003642, STX16, LOC102483497, LOC102501927, ADCY6, ITPR3, NPR2, SRC, ADCY4, NOS3, MAP2K5
Salivary secretion	Organismal Systems	0.047261	XLOC_003642, STX16, GALNT11, LOC102483497, ADCY6, ITPR3, ADCY4
Endocrine and other factor-regulated calcium reabsorption	Organismal Systems	0.047261	XLOC_003642, STX16, RUVBL2, XPNPEP1, ADCY6
cGMP-PKG signaling pathway	Environmental Information Processing	0.049559	XLOC_003642, GADD45G, XLOC_041871, BAZ1B, LOC102483497, LOC102501927, GTF2I, ADCY6, ITPR3, ATF6B, NPR2, ADCY4, NOS3

TABLE S3. The pathway enrichment of differently expressed genes (DEGs) in the $IRBP_{1197\text{-}1211}$ group

Description	Category	P_value	P_adjust	Gene name
AMPK signaling pathway	Environmental Information	0.00049529	0.131251777	ADAM23, TAF15, WDR45, WTIP, KMT2C, XLOC_053484, LOC102475736, XLOC_061060,

	Processing			SLC10A1, CREB3L2, LIPE
Methane metabolism	Metabolism	0.002479885	0.328584715	LOC102501767, BCL7C, CHMP6, WDR45
Mitophagy–yeast	Cellular Processes	0.004057935	0.358450945	LOC102488698, MAN2A2, XLOC_081167, LETM1
Phosphonate and phosphinate metabolism	Metabolism	0.014963712	0.892813468	MCRIP1, XLOC_027781
Glycerophospholipid metabolism	Metabolism	0.01742543	0.892813468	LPIN1, XLOC_007834, MCRIP1, PLA2G15, PLA2G4F, LCLAT1, XLOC_027781
Protein digestion and absorption	Organismal Systems	0.024345947	0.892813468	SLC8A1, LOC102474421, COL4A3, RHPN1, DNPEP, COL7A1
MAPK signaling pathway– fly	Environmental Information Processing	0.028411711	0.892813468	FRYL, STRN4, STS, XLOC_023302, TEC, MAP2K4, PTPN11, SRC
Biosynthesis of secondary metabolites	Metabolism	0.031052067	0.892813468	LPIN1, XLOC_007834, NFX1, LOC102501767, LOC102491763, PLA2G4F, LCLAT1, CHMP6, LOC102495698, XLOC_027781, LOC102480703, SERTAD2, TAF15, WDR45, FDPS, OGDH, MVD
Spliceosome	Genetic Information Processing	0.039902368	0.892813468	XLOC_035902, CDK10, DDX5, PQBP1, SLU7, GSKIP, XLOC_063251, XLOC_066578, LZTS3, LOC102469433,
Carbon metabolism	Metabolism	0.044057597	0.892813468	NFX1, LOC102501767, BCL7C, CHMP6, SERTAD2, WDR45, OGDH

SUPPLEMENTARY FIGURE LEGENDS

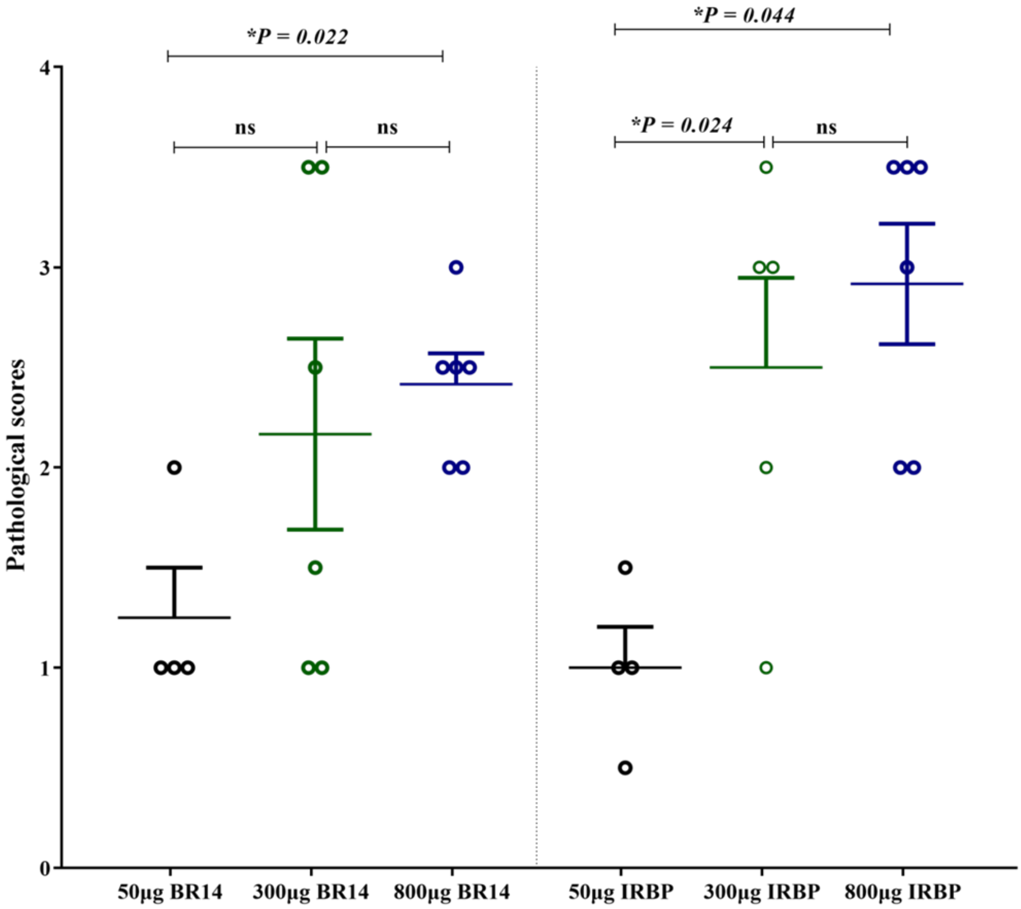

SUPPLEMENTARY FIGURE 1 Histopathological of tree shrew EAU induced by R14 or IRBP$_{1197–1211}$

Histopathological scores of the inflammatory peak in 50-μg–induced EAU were lower than those in 800-μg–induced EAU with BR14 and in 300-μg–induced and 800-μg–induced EAU with IRBP$_{1197\text{-}1211}$.

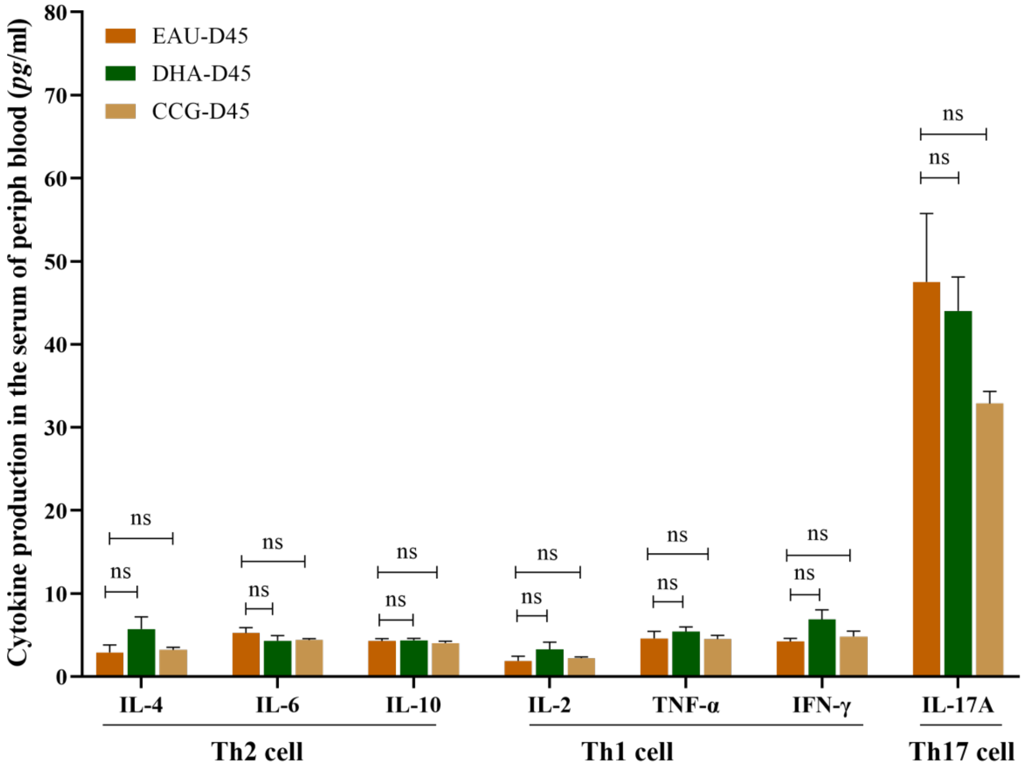

SUPPLEMENTARY FIGURE 2 Cytokine production after treatment with CCG 203769 and DHA

There was no significant change in the profiles of Th2 signature cytokines, Th1 signature cytokines, and Th17 signature cytokine in the serum of 800-μg-IRBP$_{1197–1211}$-induced EAU after treatment with CCG 203769 and DHA on day 45.
